# Integrated Multimodal Omics and Dietary Approaches for the Management of Neurodegeneration

**DOI:** 10.3390/epigenomes7030020

**Published:** 2023-09-01

**Authors:** Toshiyuki Murai, Satoru Matsuda

**Affiliations:** 1 Graduate School of Medicine, Osaka University, 2-2 Yamada-oka, Suita 565-0871, Japan; pi3kp10@outlook.jp; 2 Department of Food Science and Nutrition, Nara Women’s University, Kita-Uoya Nishimachi, Nara 630-8506, Japan

**Keywords:** Alzheimer’s disease, Parkinson’s disease, phytochemicals, dietary intervention, epigenetics, transcriptome, epigenome, proteome, multi-omics, transcriptomics, epigenomics, proteomics

## Abstract

Neurodegenerative diseases, such as Alzheimer’s disease and Parkinson’s disease, are caused by a combination of multiple events that damage neuronal function. A well-characterized biomarker of neurodegeneration is the accumulation of proteinaceous aggregates in the brain. However, the gradually worsening symptoms of neurodegenerative diseases are unlikely to be solely due to the result of a mutation in a single gene, but rather a multi-step process involving epigenetic changes. Recently, it has been suggested that a fraction of epigenetic alternations may be correlated to neurodegeneration in the brain. Unlike DNA mutations, epigenetic alterations are reversible, and therefore raise the possibilities for therapeutic intervention, including dietary modifications. Additionally, reactive oxygen species may contribute to the pathogenesis of Alzheimer’s disease and Parkinson’s disease through epigenetic alternation. Given that the antioxidant properties of plant-derived phytochemicals are likely to exhibit pleiotropic effects against ROS-mediated epigenetic alternation, dietary intervention may be promising for the management of neurodegeneration in these diseases. In this review, the state-of-the-art applications using single-cell multimodal omics approaches, including epigenetics, and dietary approaches for the identification of novel biomarkers and therapeutic approaches for the treatment of neurodegenerative diseases are discussed.

## 1. Introduction

Chronic neurodegenerative disorders are believed to be caused by a combination of multiple events that damage neuronal function [1]. Alzheimer’s disease (AD) is the most common neurodegenerative disorder, and Parkinson’s disease (PD) is the second-most common neurodegenerative disorder worldwide [1]. They are predicted to affect public health worldwide, are predominantly observed in the elderly population, and are estimated to increase threefold by the middle of the 21st century [2]. Therefore, several efforts have focused on pursuing the identification of biomarkers and causative factors in the environment, lifestyle (including diet), and genetic changes (including epigenetics) [3]. The most well-characterized biomarker for neurodegeneration is the accumulation of proteinaceous aggregates in the brain [4]. Accordingly, the definition of neurodegenerative diseases is descriptive, in that, the typical pathological feature of the aggregation of specific proteins is their hallmark, and these aggregates are determined post-mortem to characterize the disease [5]. However, the slowly progressing symptoms of neurodegenerative diseases are unlikely to be solely due to the result of a mutation in a single gene, but rather as a consequence of a multi-step process involving epigenetic changes [1].

Epigenetics is defined as the stable inheritance of a phenotype, resulting from changes in the chromosomal DNA without mutations in the nucleotide sequence [6]. A number of epigenetic research has revealed that the modification of histones accompanied by both a local and global remodeling of the chromatin structure and alternations in the transcriptional patterns are closely associated with the pathogenesis of neurodegenerative diseases, including AD and PD [7]. In particular, DNA methylation, the gain or loss of heterochromatin, and nucleolar reorganization may be involved in these diseases [8]. Thousands of epigenetic changes throughout the lifetime affect various cells and tissues and are therefore considered the key hallmarks of aging [9]. They involve alterations in the sites of DNA methylation by DNA methyltransferases; the modification of histone proteins by histone acetylases, deacetylases, methylases, and demethylases; and the structural remodeling of the chromatin [10]. In contrast to DNA mutations, epigenetic changes are theoretically reversible and therefore raise the possibility of targeting them for designing novel therapeutic interventions, including dietary alterations. The typical, age-associated epigenetic changes such as an increase in H4K16 acetylation, and H4K20 and H3K4 methylation, or a decrease in H3K9 and H3K27 methylation, constitute the characteristic age-associated epigenetic markers [10]. 

Chromatin alterations occur as the brain ages, and could therefore contribute to the development of degenerative diseases. Notably, human neurodegenerative diseases, including AD and PD are associated with drastic changes to the transcriptional profile, suggesting that epigenetic regulation might be involved in these diseases [11]. Key issues might be whether alternations to the epigenetic landscape are causal in neurodegenerative disease progression and/or initiation, or a consequence, and if so, in which specific ways [11]. Another key issue is whether dietary elements that target epigenetics can be applied to protect the brain from diseases associated with neurodegeneration [11]. With regard to this issue, there is accumulating evidence that shows nutrition can not only change epigenetic biomarker levels, but also prevent the development of late-onset AD and attenuate cognition deficit [12]. Therefore, nutrition may grow to become a preventive and even therapeutic alternative against AD, especially if combined with other antidementia interventions, brain exercise, and physical exercise [12]. In this review, the state-of-the-art applications using single-cell multi-omics approaches, including genetics and epigenetics, and dietary approaches to identify novel biomarkers and therapeutic approaches for the management of neurodegeneration are examined.

## 2. Epigenetics and Alzheimer’s Disease

AD is a common type of neurodegenerative disorder, the typical diagnostic phenotypes of which are the enhanced deposition of senile plaques composed of insoluble, neurofibrillary proteins in the brain. These aggregates mainly consist of an early accumulation amyloid-β (Aβ) protein with an abnormal form of aggregation and hyperphosphorylated tau proteins in the hippocampus that eventually lead to cognitive impairment over time [13]. The established biomarkers used at present are Aβ_1–42_, total-tau, and phosphorylated-tau in the cerebrospinal fluid (CSF). Actual therapeutic and pharmacological interventions effective in the management of AD that impede the progress of the disease have not been developed, with the only intervention being restricted to symptomatic treatments that retard the progress of the disease [14]. Therefore, the establishment of new methods effective in treating AD and the unraveling of the neurodegenerative mechanism is of significance for biomedical researchers [15]. Although the stage of the disease and the mechanism by which neurodegeneration occurs during the pathogenesis of AD have remained unresolved, many trials have been performed to clarify the pathophysiological features of neuroinflammation in AD based on the multiple parameters concerning inflammatory mediators [16]. Additionally, oxidative stress may contribute to the progression and pathogenesis of AD [17]. Oxidative stress induced in the neuronal cells was mainly attributed to the excess production of reactive oxygen species (ROS), which plays a key role in the progression of AD [18]. ROS refers to a family of ionic species continuously generated from O_2_ and scavenged within the cells. The major ROS include H_2_O_2_ (hydrogen peroxide), O_3_ (ozone), O^2−•^(superoxide anion radical), and OH^•^ (hydroxyl radical) [16]. Dysregulation of ROS contributed to the pathogenesis of neurodegenerative diseases [19]. An increased ROS production adversely affected DNA function, including its epigenetic modification related to AD [20]. In fact, an accumulating body of evidence implied that oxidative stress is the main AD risk factor by inducing the apoptosis of neurons and dysfunction of the brain at the initiation stage and throughout AD progression [21]. It is also of note that the DNA of hyper-methylated nucleotides was easily influenced by β-amyloid-promoted oxidative damage as methyl-cytosines restricted the repair of the adjacent hydroxy-guanosines [22].

## 3. Epigenetics and Parkinson’s Disease

PD is a prevalent neurodegenerative disease with the characteristics of neuropsychiatric symptoms, including depression and anxiety, existing before the onset of symptoms related to motor and movement illnesses [23]. Most patients clinically present with motor disorder and suffer from slowness of movement, rest tremors, rigidity, and disturbances in balance; the major pathophysiological features include the substantia nigra (SN)’s dopaminergic neuron loss and Lewy body depositions [23]. The dysfunction of mitochondria, which results in oxidative stress, may cause the progression of PD [24]. The dopaminergic neuron activities in the SN is critical for striatal synaptic plasticity and positive learning, and their degeneration led to an initiation of the subthalamic nucleus, that in turn amplified the excitation signals relayed to the SN [25]. Exposure to harmful mediators in the environment, such as ROS, might cause the initiation of neurodegeneration with clinical symptoms similar to those of PD [26]. Therefore, it is necessary to establish methods for ensuring the longevity of healthy neurons following an attack by ROS without using specific medications. A close association between genetics and environmental factors may determine the fate of PD, and the participation of many networks involving DNA, proteins, organelles, and neural networks may contribute to the complexity in the manifestation of symptoms [27]. Many pathogenesis-related associations have been focused on for the management of symptoms and progression of PD [27]. While dopaminergic-based treatments have been the gold standard for the symptomatic control of PD, a few requirements for the management of dopaminergic-resistant motor/non-motor symptoms remain unaddressed and for treatments altering the normal clinical course of PD [27].

Recently, much effort has been made for elucidating the epigenetics driving the alternations of gene expression associated with the pathogenesis of PD. The alternations in gene expression are a well-known cause of PD, and epigenetics is likely to play a critical role in its regulation [28]. Epigenetic regulatory mechanisms surrounding the SNCA gene, which encodes the α-synuclein (α-syn) protein, are suggested in [28]. A genome-wide epigenome study in the frontal cortex and in the blood of PD patients revealed that more than 80% of differentially methylated sites identified were hypomethylated in PD cases [28]. Among the top hits of PD hypermethylated genes was microtubule-associated protein tau (MAPT), which encodes the tau protein [28]. Therefore, an imbalanced epigenetic alternation may cause harmful effects. Environments may raise an objection for the formation and maintenance of epigenetics and may thereby fill a gap between the deeper understanding of the origin and pathogenesis of neurodegenerative diseases [29]. A recent study on genome-wide DNA methylation in brain and blood samples of PD patients revealed a characteristic methylated pattern involving a number of genes implicated in PD; thus, the functional significance of epigenetics as a regulatory factor in PD is evident, implying that peripheral blood may be a promising source for detecting DNA methylation levels in addition to brain tissue for the discovery of biomarkers associated with PD [30]. Given that PD is a disease with many related disorders—including a variety of clinical symptoms—epigenetic regulation, treatment response, and survival may possibly reflect multimodal changes, i.e., genetic, epigenetic, proteinaceous, and organellar contributions; thus, substantial improvement on diagnostic procedures may improve the success of therapeutic methods, the mechanisms of action of which may be beneficial for PD patients [31]. 

## 4. Multimodal Omics Studies for the Management of Neurodegenerative Diseases

**Figure 1:**
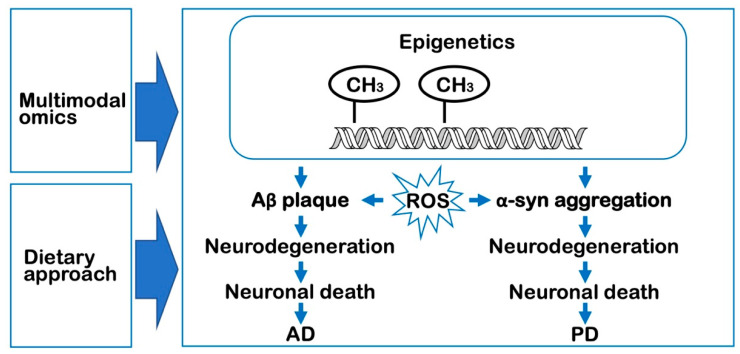
Multimodal omics and dietary approaches for the management of neurodegenerative diseases, including Alzheimer’s disease (AD) and Parkinson’s disease (PD). Reactive oxygen species (ROS) is the main AD risk factor for by inducing apoptosis of neurons and dysfunction of the brain at the initiation stage and throughout AD progression. The DNA hyper-methylation may influence the formation of amyloid-β (Aβ) plaque and α-synuclein (α-syn) aggregation, which leads to neurodegeneration and death of neurons in AD and PD, respectively. Methylation (CH_3_-) of a number of genes are also associated with PD. Unlike DNA mutations, epigenetic alterations are thought to be reversible, and therefore raise the possibilities for therapeutic intervention, including dietary modifications.

Multimodal omics approaches can be applicable for the management of neurodegenerative diseases, including AD and PD. Multiple epigenetic studies have revealed that the modification of histones accompanied by both the local and global remodeling of the chromatin structure and alternations in transcriptional patterns are closely associated with the pathogenesis of neurodegenerative diseases (Figure 1). This methodology may also be advantageous if applied to the field of epigenetics related to neurodegenerative diseases to implement novel processes for the development of sustainable production strategies toward achieving an integrative healthcare approach that combines nutrigenomics and translational medicine [32].

**Figure 2:**
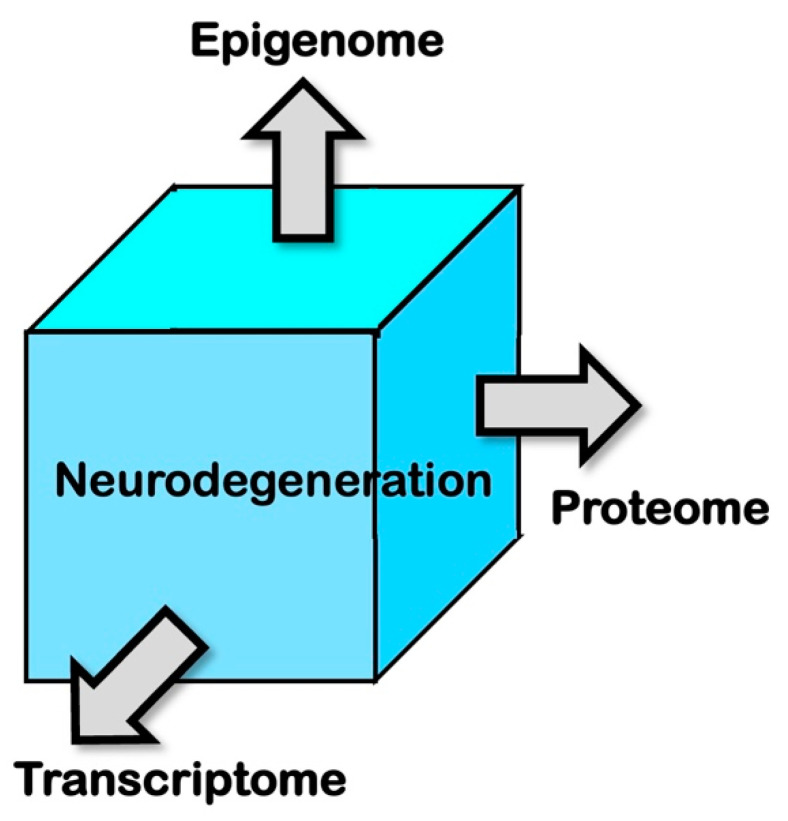
Multimodal approaches to studies in neurodegeneration utilizing the emerging technologies, including transcriptome, epigenome, and proteome analyses. Recently evolved single-cell technologies have enabled a comprehensive understanding of the biological processes orchestrated by complex networks involving biomolecular associations and genetically regulated patterns at the single-cell level. Thus, single-cell technologies can be applied for transcriptomic, epigenetic, and proteomic profiling, which may contribute to the wider areas of healthcare-related studies, especially neurodegenerative diseases, including AD and PD. The single-cell or single-nucleus transcriptome, epigenome, and proteome in humans can be harnessed into multi-modal omics analyses that are closely related to individual phenotype analysis and diagnosis and can be used in designing precision nutrition and functional medicine therapies to prevent and treat neurodegenerative diseases. Figure 2 illustrates the specific feature of multimodal approaches, so-called multi-omics, which is different from trans-omics.

In the field of molecular biology, the fourth paradigm, a data-intensive scientific discovery presented by Jim Gray in 2009, enables a comprehensive understanding of the biochemical processes conducted by complex networks involving biomolecular associations and genetically regulated patterns at the single-cell level [33] (Figure 2). Thus, single-cell technologies can be applied for transcriptomic, epigenetic, and proteomic profiling, which may contribute to the wider areas of healthcare-related studies, especially neurodegeneration, as in the case of the present review. The main focus would be on research related to single-cell/nucleus transcriptome and epigenome in humans and address the emerging techniques that bridge transcriptome and metabolome at the single-cell level. Such single-cell, multi-omics methods are closely related to individual phenotype analysis and diagnosis and can be used in designing precision nutrition and functional medicine therapies to prevent and treat neurodegenerative diseases. This field of bioinformatics has emerged as a result of the human genome project and has enabled the sequencing of DNA at a large-scale, employing high-throughput sequence methodologies [34]. These methodologies have opened the door for the systematic understanding of gene expression profiles of not only coding regions, but also the non-coding RNAs (ncRNAs), including microRNA (miRNA) or long non-coding RNAs (lncRNAs); the regulatory elements in the 5′- and 3′- untranslated regions (UTRs); and post-transcriptional RNA modification/editing events such as nucleobase modifications, including the deamination of “cytidine to uridine” and “adenosine to inosine”, additions/insertions of nucleotide(s) [35]. These herald the next-generational shift in system biology, focusing on the reconstruction of the associations for explaining the synaptic network functioning by employing machine learning techniques [36].

Many inventions and advancements have been accomplished in multi-omics approaches. Single-Cell Assay for Transposase-Accessible Chromatin sequencing (scATAC-seq) method is the method used to explore the accessibility to the genome-wide chromatin network in many cells at a single-cell resolution [35] (Table 1). Cellular Indexing of Transcriptomes and Epitopes by sequencing (CITE-seq) protocol is a single-cell technique that integrates protein measurements and transcriptomic analysis via high-throughput single-cell RNA sequencing (scRNA-seq) into an efficient, single-cell readout [37]. RNA Expression and Protein sequencing (REAP-seq) assay is a recently developed multimodal single-cell technique that enables the fine characterization of cell subtypes and functional states by measuring the expression levels of RNA and proteins [38]. A flow cytometry-based epigenomic analysis at a single-cell resolution has been developed: epigenetic landscape profiling using cytometry by time-of-flight (EpiTOF) enables us to measure the cellular levels of histone modifications by histone variants at single-cell resolution [39]. The high-dimensional and single-cell nature of these datasets allow for the creation of an epigenetic atlas with aging based on their chromatin modification profiles [40].

These emerging techniques can bridge human transcriptome and proteome at the single-cell level and these multimodal omics methodologies can be introduced for specific personal phenotyping and diagnosis for designing precise nutritional medicine. Flow cytometry has the potential to be extended on a single-cell basis to a wide range of studies, including the determination of antigen expression either on the cell surface or within cells, DNA/RNA content analysis, and functional analysis. Although flow cytometry enables the high-throughput measurement of the characteristics of one cell at a time, a high signal-to-noise ratio is a critical requirement.

Powerful methods for the characterization and sorting of cells by employing spatially and high time-resolved data, including imaged flow cytometer, have been designed [40]. However, conventional-flow cytometry-based medical diagnostics is currently seldom used in the clinical setting. The advancements in image-based cell sorting technology significantly expanded its applicability in functional omics. This methodology enabled image-based high-throughput cell sorting and multicolor imaging, ensuring a broader availability in various fields, including basic biological research and medical diagnostics, with significant potential applicability in multimodal omics readouts [41]. Flow cytometry imaging obtains multi-channel image characteristics of cells through the detectors located in image platforms that are equipped with complementary metal oxide semiconductor sensors to precisely capture images of individual cells as they flow through [41]. This intelligent, image-activated cell sorter can be integrated seamlessly with high-throughput, single-cell multi-omics analysis tools. Combining these analytical techniques with computational approaches for the analyses and integration of single-cell multi-omics data across multiple modalities can open new doors for the precise reconstruction of the genetic regulation and signal networks driving the identity and function of specific cells.

Machine learning, the utilization of trainable statistical models used to recognize patterns and predict future behavior, is a promising methodology, which is applicable for research into neurodegenerative diseases [42]. Machine learning is an application of artificial intelligence (AI) that includes comprehensive, automated analytical modeling approaches that enable machines to execute decisions by automatically learning from the available data. AI is more multifaceted than the simple statistical models being used for biological datasets. The field of bioinformatics has also emerged and gained prominence along with the human genome project and has realized the fine reading of large DNA molecules using high-throughput sequencing techniques that have opened the door for the systematic understanding of not only protein-coding gene expression profiles, but also of ncRNAs, including miRNAs and lncRNAs; the regulatory elements in the 5′- and 3′- UTRs; and post-transcriptional RNA modification/editing events, including nucleobase modifications, such as the deamination of cytidine and adenosine. This shift to the next generation in systems biology aims to reconstruct the interactions for explaining the synergistic networks by introducing machine learning [43].

Recently, a genome-wide association study employing single-cell multimodal approach on neurodegenerative diseases has provided a high-resolution epigenetic characterization of the role of inherited noncoding variation in AD and PD [44]. In that study, single-cell ATAC-seq (scATAC-seq) data over a machine learning classifier predicted putative functional SNPs driving the association with neurodegenerative diseases [44]. The profiling of single-cell chromatin accessibility landscapes and three-dimensional chromatin interactions of diverse adult brain regions across a cohort of cognitively healthy individuals has enabled the prediction of dozens of functional SNPs for AD and PD, nominating target genes and cell types for previously orphaned loci from genome-wide association studies [44]. The study expands the understanding of inherited variation and provides a roadmap for the epigenomic dissection of causal regulatory variation in disease [44]. Another integrated multimodal omics approach suggested that AD involves a reconfiguration of the epigenome, wherein H3K27ac and H3K9ac affect disease pathways by dysregulating transcription– and chromatin–gene feedback loops [45]. The identification of this process highlights potential epigenetic strategies for early stage disease treatment [45].

## 5. Dietary Approaches for the Management of Neurodegeneration

The mutual relation between diet/nutrition and the immune system plays an important role in the maintenance of the human body. A systematic determination of the full range/variety of cells has been a challenge due to limitations of analytical methods that have restricted the parameters that can be determined. Recently, the single-cell techniques have overwhelmed these limitations with their high-throughput properties [46]. The interactions also contribute to the progression of diseases, including neurodegenerative diseases. Therefore, along with these large international projects, a healthcare-based approach integrating single-cell techniques may offer deeper insights into the current state of studies on nutritional and molecular medicine and serve to discover and develop new clinical interventions for the treatments of diseases shortly [47]. 

Accumulating evidence suggests that a variety of dietary plant phytochemicals exhibit neuroprotective effects. For example, resveratrol improved overall cognitive performance in postmenopausal women [48]. Another randomized phase II trial of resveratrol in individuals with mild to moderate AD revealed that Aβ40 in CSF and plasma Aβ40 levels declined more in the placebo group than the resveratrol-treated group [49]. Resveratrol supplementation prevents cognitive decline by restoring the epigenetic landscape as well as synaptic plasticity [50]. Anthocyanin, richly present in blueberry, inhibited Aβ fibrillation and a reduced ROS production in microglial cells [51]. Hydroxytyrosol found in diverse vegetable species exhibited an antioxidant effect in a PD model [52]. Ginsenoside and paeoniflorin decreased α-syn fibrillation and accumulation, respectively [53,54] (Table 2). A variety of other dietary compounds possess neuroprotective properties, and may act on several cellular targets to prevent the development of PD or to attenuate the progress of the disease [55]. Notably, many clinical and pre-clinical studies have reported protective effects of certain dietary micronutrients for PD [55]. The candidate micronutrients for therapeutic use in PD have been summarized in the literature [55]. Further well-designed clinical studies are needed to evaluate the therapeutic benefits of these dietary phytochemicals as promising agents in the management of PD.

## 6. Conclusions and Future Directions

Neurodegeneration during the course of AD and PD pathogenesis are caused by a combination of multiple events that damage neuronal function. Although a well-characterized biomarker of such neurodegeneration is the accumulation of proteinaceous aggregates in the brain, the gradually worsening symptoms of neurodegenerative diseases are unlikely to be solely due to the result of a mutation in a single gene, but rather a multi-step process involving epigenetic changes. 

It is of note that sex bias and differences should be considered. In fact, an important recent report clearly indicates the pivotal role of epigenetics on health maintenance that is associated with the health status, and also to the sex difference of the patient [56,57]. Men were more frequently classified as hippocampal sparing AD, whereas limbic predominant AD was more prevalent in women [58]. However, men and women do not differ in the frequency of MAPT haplotype or apolipoprotein E (APOE) genotype [58]. Sex differences in incidence, prevalence, and in the severity of neurodegenerative diseases, as well as sex-biased transcriptomics profiling in different tissues and sex-specific effects upon immunity, microbiota, inflammation, and epigenome are possible factors [58,59,60]. The estimation of these factors has been summarized elsewhere [58,60], which should be evaluated as a comprehensive and integrated approach to neurodegeneration in order to suggest a proper diet as a therapy. Although currently available therapeutic methods can slow down the progression of AD, the therapeutic approach for the management of AD is not fully effective to date [61]. Epigenetic modulators such as histone deacetylase (HDAC) inhibitors have demonstrated therapeutic potential and have been approved for the management of AD [61]. However, these are also in their initial stages and extensive research needs to be conducted to derive conclusive evidence for the benefit of these strategies for AD management [61].

Technology serves as a strong partner in biochemistry and molecular medicine, allowing for discoveries that were previously beyond the reach of researchers. Path-breaking exploration is possible with the improvement and innovation of novel techniques/tools. Advances in technology have also been inspired by the systems and processes of organisms or the need to establish a novel methodology to solve a biological problem. Such a relationship is powerful when applied to the synergistic exploration of fundamental problems in biochemistry and molecular medicine. Currently, improvements in the visualization and manipulation of biological systems as well as analyzing and integrating big data and the scope of the discovery are important. Studies on neurodegenerative diseases have developed through the use of advanced technologies; recently, improved sequencing techniques and single-cell/single-nucleus technology enabled the analysis of multiple human-associated species for transcriptomic studies. These methodologies have become routine in recent studies concerning neurodegenerative diseases; single-cell technologies have revolutionized the studies with much improvement in the throughput. These analytical methods used for neurodegenerative diseases overcome one of the challenges that differ among individuals and epigenetic complexity. Unlike DNA mutations, epigenetic alterations are reversible, and therefore raise the possibilities for therapeutic intervention, including dietary modifications. Additionally, reactive oxygen species may contribute to the pathogenesis of Alzheimer’s disease and Parkinson’s disease. Given that the antioxidant properties of plant-derived phytochemicals are likely to exhibit pleiotropic effects against ROS-mediated epigenetic alternation, dietary intervention may be promising for the management of neurodegeneration in these diseases. Thus, the recent innovations which include single-cell methods have much potential for applicability in the analysis of neurodegeneration.

## Figures and Tables

**Table 1:** Representative single-cell approaches applicable to multimodal analysis of neurodegenerative diseases.

Methods	Targets	References
scATAC-seq ^1^	Epigenome	[35]
CITE-seq ^2^	Transcriptome and proteome	[37]
REAP-seq ^3^	Transcriptome and proteome	[38]
EpiTOF ^4^	Epigenome	[39]

^1^ scATAC-seq: Single-Cell Assay for Transposase-Accessible Chromatin sequencing. ^2^ CITE-seq: Cellular Indexing of Transcriptomes and Epitopes by sequencing. ^3^ REAP-seq: RNA Expression and Protein sequencing. ^4^ EpiTOF: epigenetic landscape profiling using flow cytometry by time-of-flight.

**Table 2:** The potential effects of dietary phytochemicals in neurodegenerative diseases.

Phytochemicals	Effects	References
Resveratrol	Enhance cognition	[48]
Resveratrol	Decrease in Aβ level	[49]
Resveratrol	Epigenetics	[50]
Anthocyanin	Inhibit Aβ fibrillation	[51]
Hydroxytyrosol	Antioxidant effect in PD	[52]
Ginsenoside	Decrease in α-syn fibrillation	[53]
Paeoniflorin	Decrease in α-syn accumulation	[54]

## Data Availability

No new data were obtained or analyzed in this study. Data sharing does not apply to this article.

## Abbreviations

Aβ	amyloid-β
AD	Alzheimer’s disease
AI	artificial intelligence
APOE	apolipoprotein E
ATAC-seq	Assay for Transposase-Accessible Chromatin sequencing
CITE-seq	Cellular Indexing of Transcriptomes and Epitopes by sequencing
CSF	cerebrospinal fluid
HDAC	histone deacetylase
lncRNA	long non-coding RNA
MAPT	microtubule-associated protein tau
miRNA	microRNA
ncRNA	non-coding RNA
PD	Parkinson’s disease
REAP-seq	RNA Expression and Protein sequencing
ROS	reactive oxygen species
scRNA-seq	single-cell RNA sequencing
scATAC-seq	Single-Cell Assay for Transposase-Accessible Chromatin sequencing
SN	substantia nigra
α-syn	α-synuclein
t-SNE	t-stochastic neighbor embedding
UTR	untranslated region
